# Emergency management: angle-closure glaucoma

**Published:** 2018-11-09

**Authors:** Desirée Murray

**Affiliations:** 1 Lecturer in Ophthalmology: Department of Clinical Surgical Sciences, The University of the West Indies, Mount Hope, Trinidad and Tobago, West Indies.

**Acute angle-closure glaucoma is an ophthalmic emergency as it can lead to irreversible blindness if not identified and treated immediately.**

## Presentation

The patient may complain of a painful red eye, headache, blurred vision, haloes, nausea, vomiting and abdominal pain (sometimes misdiagnosed as gastroenteritis). Precipitating factors include dim light and certain drugs (e.g., bronchodilators, cough mixtures, cold and flu medication, antidepressants, antihistamines and anticonvulsants).

## Examination

Examination findings include conjunctival injection around the cornea (red eye), mid-dilated nonreactive pupil, corneal haze, diminished red reflex and a hard globe, with intraocular pressure (IOP) of between 50 and 80 mmHg. Central retinal artery and central retinal vein occlusion may also occur.

**Figure 1:**
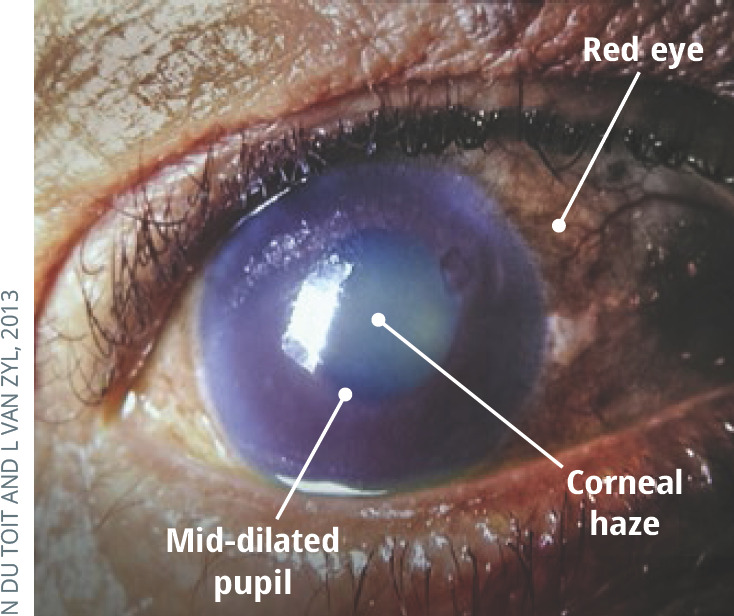
Clinical findings in acute angle-closure glaucoma.

## Protocol

**Treatment goal:** immediate lowering of IOP and alleviation of inflammation, pain, nausea.

1. **Relieve pupil block.** Ask the patient to lie down on her or his back. This improves the lens position (it will be more posterior) and thereby relieves pupil block.
2. **Lower IOP.** Give acetazolamide 500 mg, preferably intravenously or orally, if intravenous is not available or if the patient is not nauseated. Instil topical glaucoma medications (beta blockers, alpha agonists and prostaglandin analogues).
3. **Reduce pain** by giving analgesics and **reduce inflammation** by instilling topical steroids.
4. **Reduce nausea and vomiting.** Give anti-emetics.

After approximately 1 hour, the decrease in IOP should improve blood supply to the iris and make it more responsive to pilocarpine.

**Instil pilocarpine** (2% or 4% eye drops) in two doses, spaced 15 minutes apart. If IOP remains dangerously elevated after the second dose of pilocarpine, consider giving hyperosmotic agents such as glycerol, isosorbide or mannitol. **Extreme caution is advised in patients with cardiovascular conditions and renal impairment, as the side effects can be life-threatening. Glycerol is contraindicated in patients with diabetes.**

## Referral and treatment

Once the patient is stabilised, refer her or him to an ophthalmologist immediately.

Because the lens plays a major role in the mechanism of acute angle-closure glaucoma, cataract extraction can be considered as a definitive treatment for patients with co-existing cataract and presenting IOP >55 mmHg.^1^ After the acute attack is successfully treated with medication, the cataract is replaced by a thinner artificial lens implant, thereby relieving the pupil block.

**Figure 2:**
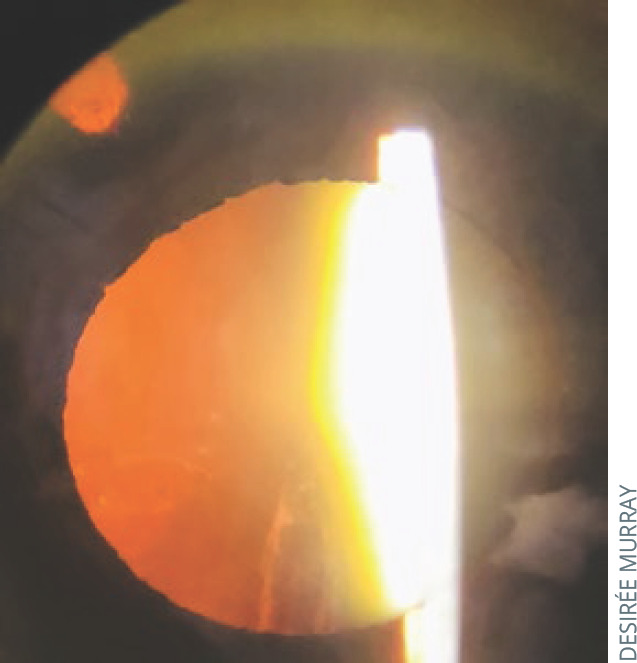
Peripheral iridotomy

In other patients, the basis of treatment is an iridotomy: the creation of a hole in the peripheral iris (Figure 2), either surgically or using a laser. This bypasses the pupil block and reestablishes flow from the posterior to the anterior chamber. If the other eye is at risk, iridotomy is performed in both eyes.

## How to prepare for this emergency

Put together an acute angle-closure glaucoma emergency kit containing all the medication (see panel),^2^ needles and syringes that may be needed. Include a copy of the treatment protocol and the contact details of the nearest ophthalmologist. This will ensure that you and your team are prepared. Check expiry dates regularly as this sight-threatening emergency is uncommon. The storage container should be clearly labelled and kept in the emergency room for easy access. Every team member must know where the kit is stored and be familiar with its contents.

**Emergency kit: medication**

- Intravenous acetazolamide 500 mg, provided as a sterile powder requiring reconstitution (or oral acetazolamide if intravenous is unavailable)
- Hyperosmotic agents
  - Oral glycerol 1.0–1.5 g/kg (*contraindicated in patients with diabetes*)
  - Oral isosorbide 1.5–2.0 g/kg (*as an alternative in patients with diabetes*)
  - Intravenous mannitol 1–2 g/kg (500 ml of 20%)
- Topical steroids (prednisolone)
- Topical glaucoma drugs
  - Beta blocker (timolol)
  - Alpha 2 agonist (brimonidine, apraclonidine)
  - Prostaglandin analogue (latanoprost, travoprost, bimatoprost)
- Topical pilocarpine 2% or 4%
- Non-oral analgesics (or oral if other routes are not available)
- Non-oral anti-emetics (or oral if other routes are not available)
- Contact details of the nearest ophthalmologist (on-site or off-site) for emergency referral.

## References

[1] Lam DSC Leung DYL Tham CCY Li FCH Kwong YYY Chiu TYH Fan DSP. Randomized trial of early phacoemulsification versus peripheral iridotomy to prevent intraocular pressure rise after acute primary angle closure. Ophthalmology 2008;115(7):1134–1140. 18164064 10.1016/j.ophtha.2007.10.033

[2] IAPB Essential List for Glaucoma. Available at **https://iapb.standardlist.org/wp-content/uploads/2017/04/IAPB_EL_for_Glaucoma_2017.pdf** (accessed 10 June, 2018).

